# Lignin-Based Hydrogels: Synthesis and Applications

**DOI:** 10.3390/polym12010081

**Published:** 2020-01-03

**Authors:** Diana Rico-García, Leire Ruiz-Rubio, Leyre Pérez-Alvarez, Saira L. Hernández-Olmos, Guillermo L. Guerrero-Ramírez, José Luis Vilas-Vilela

**Affiliations:** 1 Chemistry Department, University Center of Exact Sciences and Engineering, University of Guadalajara, 44430 Guadalajara, Mexico; dianargiq@gmail.com (D.R.-G.); saira.hernandez.olmos@gmail.com (S.L.H.-O.); guillermo.guerrero@red.cucei.udg.mx (G.L.G.-R.); 2 Macromolecular Chemistry Group (LQM), Physical Chemistry Department, Faculty of Science and Technology, University of the Basque Country, 48940 Leioa, Spain; leyre.perez@ehu.eus (L.P.-A.);; 3 BCMaterials, Basque Center for Materials, Applications and Nanostructures, UPV/EHU Science Park, 48940 Leioa, Spain

**Keywords:** lignin, hydrogels, renewable sources

## Abstract

Polymers obtained from biomass are an interesting alternative to petro-based polymers due to their low cost of production, biocompatibility, and biodegradability. This is the case of lignin, which is the second most abundant biopolymer in plants. As a consequence, the exploitation of lignin for the production of new materials with improved properties is currently considered as one of the main challenging issues, especially for the paper industry. Regarding its chemical structure, lignin is a crosslinked polymer that contains many functional hydrophilic and active groups, such as hydroxyls, carbonyls and methoxyls, which provides a great potential to be employed in the synthesis of biodegradable hydrogels, materials that are recognized for their interesting applicability in biomedicine, soil and water treatment, and agriculture, among others. This work describes the main methods for the preparation of lignin-based hydrogels reported in the last years, based on the chemical and/or physical interaction with polymers widely used in hydrogels formulations. Furthermore, herein are also reviewed the current applications of lignin hydrogels as stimuli-responsive materials, flexible supercapacitors, and wearable electronics for biomedical and water remediation applications.

## 1. Introduction

As a result of the gradual depletion of fossil resources and the constant increase in the world’s population, the interest in finding an alternative, clean, and globally available resource has arisen. Biomass is the second most abundant material and, due to its composition, it has the ability to replace many petroleum products. The plant biomass consists of 40–45 wt% of cellulose, 25–35 wt% of hemicellulose, 15–30 wt% lignin, and up to 10 wt% of inorganic components [1]. Among the three main components, cellulose and hemicellulose are the ones with the highest added value owing to their broad applications in industry, while lignin is considered inexpensive, so currently, 98% is burned to generate energy in production plants and the remaining 2% is used in the manufacture of livestock feed, vanillin, fillers in cements, in the manufacture of carbon fibers, as well as complement in agglomerates and adhesives [2,3,4,5,6,7]

Lignin plays a fundamental role in plants, in that it helps the cell wall and internal fibers to transport water and nutrients, protects them from microbial attack, and provides mechanical support to the plant. Previous studies have determined the amount of lignin in different plants, being the coconut fiber with 45% the plant that contains the highest percentage of lignin [8], follow by rice husk with 35% [9] and wood containing 10%–35% lignin [10]. However, even though coconut fiber is the one that contains the highest amount of lignin, its consumption and production is much lower than wood. Wood is the main raw material in the paper industry and consequently, an important by-product of this industry is lignin.

Two types of wood are normally used for paper production, hardwood and softwood. Hardwood is usually obtained from pine, fir, spruce and larch trees and softwood is obtained from eucalyptus, poplar and birch trees. Although both types of wood are used to produce different types of paper, the most used is softwood. This has an advantage for obtaining lignin, since this type of wood contains a higher percentage of lignin and, consequently, makes lignin an extremely abundant and inexpensive product.

### 1.1. Lignin

**Figure 1:**
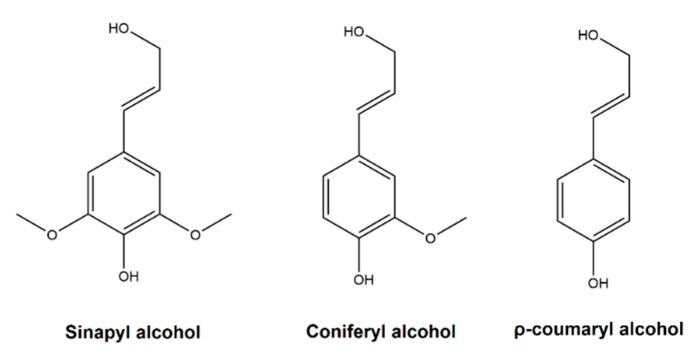
The three monolignols considered as the building blocks of lignin.

Chemically, lignin is a highly branched, amorphous biomacromolecule that normally presents molecular masses between 1000 and 20,000 g/mol. At present, it does not exist a defined lignin structure, since the determination of the lignin structure is extremely complex. The structure of lignin is constituted by different structures and repeated units, which depend on the species, location, growth duration of the plant, and the extraction process used to separate it from cellulose and hemicellulose. However, it is known that the structure of the lignin, regardless the plant and the extraction process, is composed mainly of three different phenylpropane monomers called sinapyl alcohol, coniferyl alcohol, and ρ-coumaryl alcohol [11] which give rise to units of p’-hydroxyphenyl (H), guaiacyl (G) and syringyl (S). Figure 1 shows the three monolignols that are considered the building blocks of lignin.

**Figure 2:**
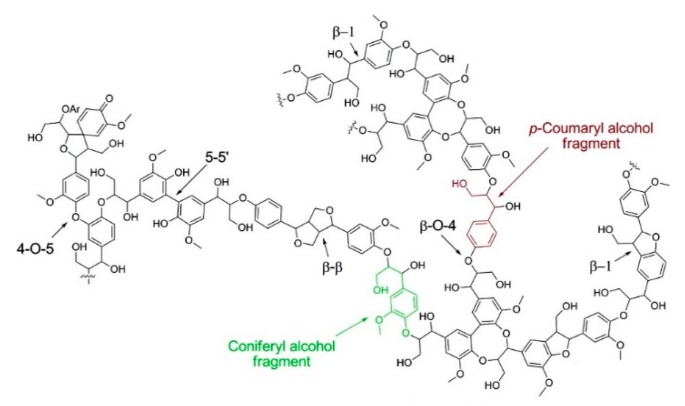
Structure of softwood lignin. Reprinted with permission from Zakzeski et al. [5]. Copyright (2010) American Chemical Society.

These subunits contain many different functional groups, such as hydroxyls (20% to 30%), carbonyls (20%) and methoxyls (90%–95%), which are active sites for further chemical modification of lignin [12]. Figure 2 shows the structure of lignin from softwood.

The heterogeneous nature of wood means that there is not currently available method for the quantitative isolation of lignin without the risk of structurally modifying it during the process. As above mentioned, the main source for obtaining lignin is the paper industry where enzymatic, chemical and mechanical processes are used. The different processes produce different types of lignins [13], for example, alkali lignin, which is mainly obtained from the kraft process [14], hydrolytic lignin obtained by enzymatic hydrolysis [15], organosolv lignin by organosolv process [16], and lignosulfonates obtained by the sulfite process [3].

#### 1.1.1. Kraft Process

The kraft process for obtaining lignin consists in two consecutive steps. In the first step, an aqueous solution of sodium hydroxide and sodium sulfate react with the wood in a digester, a container at high pressure, at around 170 °C [12,17]. In a second step, the kraft lignin is obtained [18]. The kraft process is highly effective and its main advantage is the recovery and reuse of salts. The principal disadvantage of this process are the exhausted bad odors produced by the emission of thiols and sulfides during the reactions. Due to this extraction reaction, the kraft lignin contains in its structure from 1 to 3 wt% of sulphur groups. Therefore, in order to increase its applications, this kind of lignin is often sulfonated to make it soluble in water. Commonly, kraft lignin is used in the manufacture of polyurethanes and epoxy resins [6,19].

#### 1.1.2. Sulphite Process

Lignins obtained from sulfite pulp processes are called lignosulfonates and they are produced by a reaction with sulphurous acid and a sulfite salt containing magnesium, calcium, sodium, or ammonium at different pH levels [3]. Lignosulfonates present sulfonation degree, this increases the water solubility [3]. This type of lignin is generally intended to be used as animal feed, pesticides, surfactants, oil extraction additives, stabilizers in colloidal suspensions and plasticizers in concrete mixtures, however, the product with the greatest added-value in the market is vanillin [7].

#### 1.1.3. Organosolv Process

Another lignin extraction process is the organosolv treatment, in which organic solvents are used to separate the lignin from the cellulose. In this process, a rotary digester is generally used in which ethanol and sulfuric acid are added to the wood dust. The main disadvantage of this process is that it can only be used to extract lignin from hardwoods, since the percentage of lignin present in this type of wood lower than softwood, the amount of lignin obtained is lower than in other processes [16,20]. This type of process produces lignins with low quantities of sulphur and their main use is in the manufacture of adhesives and phenolic resins [2].

#### 1.1.4. Enzymatic Hydrolysis Process

Lignin could also be obtained by an enzymatic hydrolysis of the wood carbohydrates by cellulolytic enzymes leaving lignin as an insoluble residue. In this process, the biomass is subjected to successive enzymatic treatments to force the complete hydrolysis and dissolution of the carbohydrates of the cellulose and hemicellulose, increasing the amount of insoluble recovered lignin. Usually, obtained lignin samples contain 65–80% lignin, 7–8% carbohydrates, and impurities, often proteins generated during enzymatic treatment [15]. This type of lignin is generally intended to be used as a binder, dispersant, and emulsifier [21].

In addition to the abundant availability and inexpensiveness, another interesting property of the lignin is the biodegradability, which is a highly desirable property for lignin-based materials. In nature, the degradation of lignin is caused by certain fungi, as well as, several bacteria. Fungi are known to be more efficient at breaking down lignin than bacteria, in which fragmentation is slower and more limited. The microorganisms that degrade lignin are formed by unique extracellular enzyme systems that break down lignin through radical-based oxidative reactions. These microorganisms produce a set of different combinations of enzymes with multiple isoenzymes and isoforms by responding to various environmental stimuli such as nutrient availability, oxygen concentration, and temperature, which are believed to allow effective decomposition of lignin into lignocellulosic biomass [22]. Microbial degradation of lignin has not been studied extensively in organisms other than fungi, but there are reports of bacteria that can break down lignin. These lignin-degrading bacteria represent mainly three classes: actinomycetes, α-proteobacteria and γ-proteobacteria [23].

With respect to the chemical valorization of lignin. One of the challenges that researchers have sought to overcome is the depolymerization of lignin in order to obtain low molecular weight molecules that can be used as substitutes for other synthetic molecules currently used. The main drawback to lignin depolymerization is the lignin polyphenolic structure, which is chemically stable and difficult to modify, requiring difficult experimental conditions to depolymerize. Among the methods studied for lignin fragmentation pyrolysis, enzymatic oxidation, hydrolysis and hydrogenation are worth being highlighted [24].

Currently research has redirected its search to improve the physicochemical properties of lignin by chemically modifying specific active sites in the structure of the lignin in order to broaden its range of applications. These modifications consist of increasing the reactivity of hydroxyl groups or changing the nature of the active chemical sites of the lignin structure [25]. Amination, hydroxyalkylation, nitration, halogenation and sulfomethylation are examples of specific chemical modifications that have been made to lignin [26].

A specific chemical modification that deserves special attention is the modification of hydroxyl groups, since they are groups with high reactivity and if modified, they can significantly affect the reactivity of the lignin. Lignin has two types of hydroxyl in its structure; phenolic hydroxyl and aliphatic hydroxyl groups in the positions C-α and C-γ in the side chain. This type of modification affects the hydroxyl groups and results in the formation of polyol lignin derivatives, which improve in some cases the solubility of the lignin. That is, the polyols could take part in several reactions such as esterification, urethanization, phenylation and etherification, in which demethylated lignin, lignin-based esters, lignin-based polyurethanes, phenol-formaldehyde resins and lignin-based polyethers are formed, respectively. All these reactions are those mainly used to modify the hydroxyl groups of the lignin molecule [27].

Although lignin currently has direct applications in the polymer industry, the main drawback is that it can only be incorporated in small quantities, as its thermal degradation and mechanical properties must be considered. Because of this, the chemical modification of lignin seems to be the best way to use this renewable product as a starting material for the chemical synthesis of better and new materials, such as composites or hydrogels [28,29,30].

### 1.2. Hydrogels

Hydrogels are crosslinked hydrophilic polymeric materials in the form of a three-dimensional network of natural or synthetic origin [31,32], obtained by polymerization and simultaneous crosslinking of one or more monomers. Hydrogels are materials that stand out for their swelling properties, morphology and resistance to compression. These characteristics are granted based on their chemical composition and synthesis conditions.

The main synthesis route for hydrogels is polymerization in solution, in which, in addition to the monomers, there is a crosslinking agent that will give to the hydrogel the main characteristic of insolubility in water. Regardless of the type of polymerization and the monomers used, it is necessary to use a polymerization reaction trigger or initiator. The initiation systems that can be used are free radicals, temperature, ionic, gamma radiation or redox pair.

Other interesting properties of these materials are their ability to control the diffusion process, response to changes in ionic strength, pH and/or temperature, and the ability to trap chemical species through functional polar groups that interact selectively and strongly with those species [29].

Hydrogels can be classified into two types chemical or physical, depending on the nature of the interactions that form the three-dimensional network. Chemical hydrogels are those in which the network is formed through covalent bonds and this gives to the final product different characteristics such as more resistant mechanical properties. Unlike chemical hydrogels, physical hydrogels are made up of weaker interaction generally hydrogen bonds, ionic forces, hydrophobic forces or van der Waals interaction.

In most cases, a single monomer does not provide good mechanical properties and high water retention, so it is necessary to resort to copolymerization in order to obtain a better performance of these properties [33]. The applications of hydrogels are extensive and variable, and the most relevant are in the capture of heavy metal ions, medical devices, therapeutic applications and in the controlled release of drugs [34].

## 2. Synthesis of Lignin-Based Hydrogels

Modification of lignin has allowed for the preparation of highly valuable lignin derivatives. This is the case of lignin-based hydrogels, that are recognized as a more sustainable and environmental-friendly alternative to synthetic hydrogels.

Due to the attractive properties of lignin such as biodegradability, biocompatibility, non-toxicity and sustainability promotion, it is an excellent candidate for being part of basic formulations of hydrogels. The presence of hydrophilic functional groups on the chemical structure of lignin endorses its role as polymer for hydrogels preparation, and justifies its applicability in agriculture, petroleum oil drilling, water and soil pollution treatment, biomedicine, and as absorbent product. However, as a consequence of its complex structure and rigid properties, despite being the second most abundant naturally macromolecule in nature, limited works have been reported on lignin hydrogels.

Lignin has been included along the last decades in different polymeric networks by mean of physical interactions or chemical reactions promoting hydrogels structure.

### 2.1. Lignin Hydrogels by Physical Interaction

Physical mixture consists of the simple dispersion or blending of lignin with other different polymer/s, acting lignin as biomass when it is immobilized within a polymer matrix. This approach tries to exploit the basic role of lignin in nature that is, being the structural strength of plants, in order to improve the mechanical properties of preformed polymeric networks. However, the thus obtained semi-interpenetrating polymeric networks (IPNs) have been scarcely investigated. For example, Peñaranda et al. [35] polymerized and crosslinked acrylamide (AAm) by free radical reaction with the crosslinking agent N,N′-methylene-bis-acrylamide (NMBA) in the presence of lignin (different combination with starch and peat were also analyzed). Scanning electron microscopy (SEM) and infrared spectroscopy (FTIR) studies revealed an IPN structure in which lignin was found only dispersed in the polyacrylamide crosslinked network. Prepared IPN hydrogels were employed for the adsorption of Cu(II) and Ni(II) metal ions for wastewater treatment purposes.

**Figure 3:**
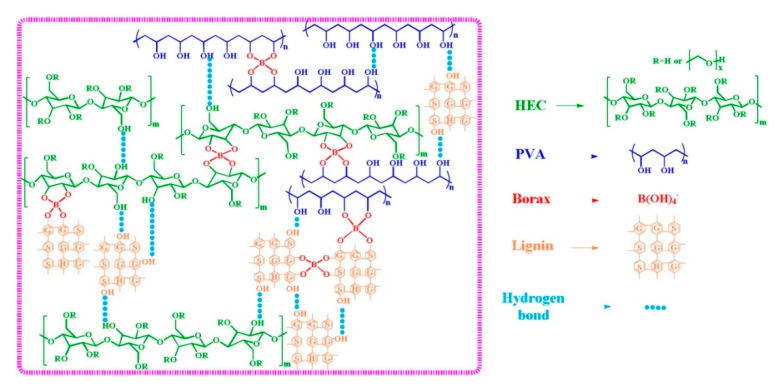
Schematic representation of the structure mechanism of PVA/HEC/lignin hydrogels with valuable self-healing properties Reproduced with permission from Huang et al. [37] Copyright © (2019) Elsevier.

The abundant polar sites on lignin’s backbone can be also employed for physical crosslinking of hydrophilic polymers by H-bonding. This was the case of the hydrogels prepared for Oveissi et al. [36] in which lignin was added to polyurethane hydrogels and it was confirmed that hydrogen bonding led to improve the mechanical properties and the processability of the final hydrogels, while swelling properties remained similar. H-bonding were also established on hydroxyethyl cellulose (HEC) and polyvinyl alcohol (PVA) hydrogels physically crosslinked with borax, between lignin and cited polymers (Figure 3). Lignin uniformly dispersed within the network showed to act as plasticizer, increasing polymer chains mobility and consequently, leading to high stretchability [37]. Hydrogels showed self-healing capability due to the dynamic hydrogen bonding between HEC, lignin, PVA, and reversible diol-borax complexation.

Ionotropic gelation was also observed between alkaline lignin and chitosan leading to physical hydrogels by simple mixing of both polymers [38]. This gelation was a consequence of the electrostatic interactions between phenoxide anions of lignin and the protonated cationic amine groups of chitosan. The viscoelastic properties of these gels were comparable to those of similar chitosan physical gels, however, a plasticization effect due to the action of alkali lignin was observed.

Chitosan and polyvinylalcohol formed hydrogels in the presence of the lignin obtained as a by–product of sulfite papermaking pulp (sulfonation degree of 11.6%). FTIR and SEM microphotographs analyses pointed out the role of lignin as macromolecular crosslinking agent by hydrogen bonding between lignin and PVA hydroxyl groups, and by the electrostatic interactions between the cationic amino group of chitosan and the sulfonic moiety of lignin [39].

### 2.2. Lignin Hydrogels by Chemical Interaction

With regard to the chemical reactivity of lignin, it is noteworthy that it is also based on the specific functional groups present on its chemical structure that support its hydrophilicity, as well as on the structural modifications induced by the extraction methodology. Lignin displays plenty phenolic and aliphatic hydroxyl groups on the side chain. These hydroxyl groups enable chemical reaction traditionally via hydroxymethylation and epoxidation by reaction with formaldehyde and epichlorohydrin, respectively, leading to crosslinked structures. Certainly, lignin has been included in the field of phenolic and epoxy resins as a substitute for phenol due to the similarity between phenol and lignin [40].

**Figure 4:**
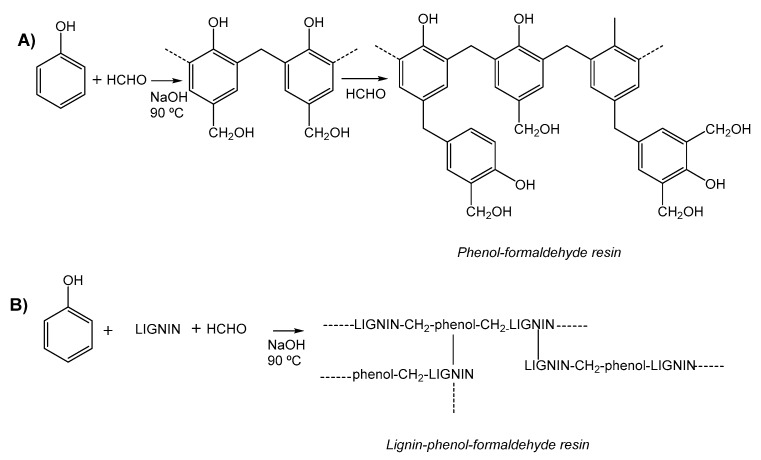
(**A**) Schematic representation of the synthesis of general phenol-formaldehyde resins. (**B**) Schematic representation of the synthesis of lignin-phenol-formaldehyde resins.

When lignin reacts with formaldehyde its hydroxymethylation takes place and introduced OH groups, after reaction with phenol moieties led to lignin based polymerized networks (Figure 4B), like phenol react with formaldehyde in alkaline medium forming the well-known phenol-formaldehyde resins (Figure 4A). Hydroxymethyl groups are introduced in the lignin’s reactive positions (at a free ortho position of the phenolic hydroxyl group of lignin) through the electrophilic substitution of formaldehyde (Figure 4), and as in the simple case of phenol-formaldehyde, crosslinked structures are formed [41].

Consequently, lignin has been employed as total or partial renewable substitute for phenol in the synthesis of phenol–formaldehyde-based resins. Since lignin displays a similar structure with aromatic hydroxyl groups it presents similar crosslinking ability than phenol–formaldehyde resins (Figure 4). However, lignin reactivity with formaldehyde is restricted by steric hindrances, usually leading to phase separation for high lignin/phenol ratios. For this reason, the chemical modification of lignin is an alternative to promote reactivity and it has been carried out by acid hydrolysis, acetosolv, and organosolv processes [42].

Lignin and phenols were employed as inexpensive raw materials for preparing gels by Grishechko et al. [41] who analyzed the changes of obtained porous structures according to the ratio lignin-phenol-formaldehyde and the employed drying method. The same authors also added tannin to prepare lignin gels from lignin-tannin-formaldehyde by thermo-chemical activation. Previously, Chen et al. [40], instead of phenol, employed resorcinol to prepare gels (aerogels and cryogels) of lignin. These lignin–phenol–resorcinol resins were additionally crosslinked by the action of the crosslinking agent glutaraldehyde under mild conditions (3 days at 25 °C). Degradation of crosslinked hydrogels by some microorganisms was investigated, showing high performance with the fungus *Flammulina velutipes*.

**Figure 5:**
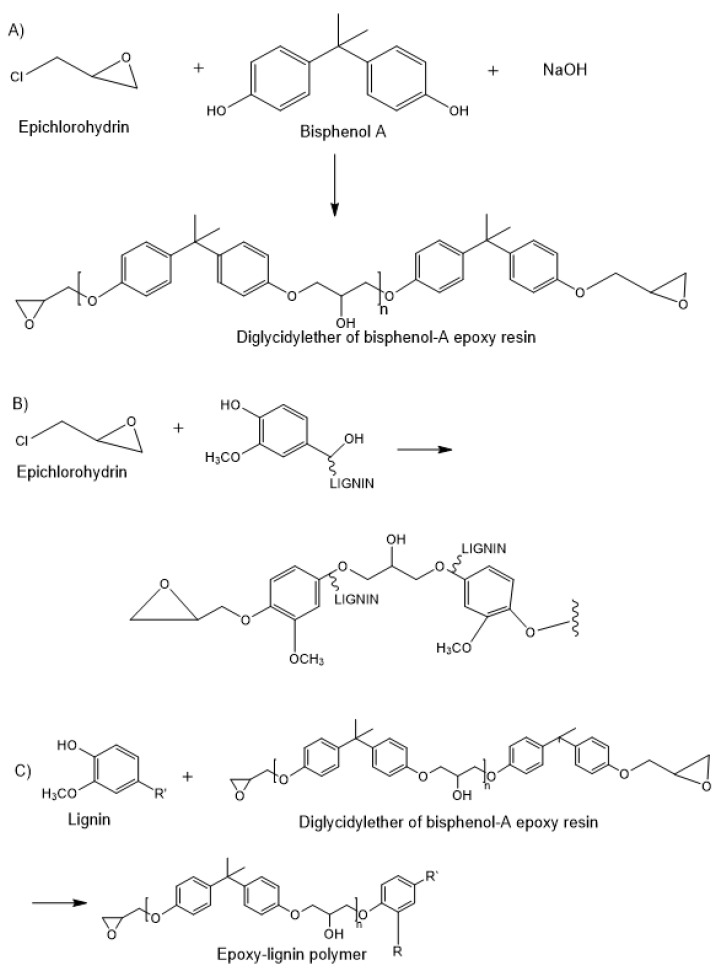
Schematic representation of the synthesis of (**A**) general bisphenol A-epoxy resins, (**B**) crosslinking of lignin with epichlorohydrin and (**C**) lignin- bisphenol A epoxi-resins.

Regarding lignin-based epoxy resins, an epoxidation reaction takes place by reaction with epichlorohydrin in alkali conditions, using lignin, that has been demonstrated to be a promising bio-replacement of bisphenol-A in the production of epoxy resins with acceptable properties (Figure 5) [43].

Ligning-based epoxy resins have been developed not by chemical reaction, but by blending lignin with petroleum based epoxy resin, to prepare composite materials. In these cases, lignin reacts with the epoxy resin upon curing. However, the simple blending approach restricts epoxy resins substitution by lignin (<20–30 wt%) [43]. Yin et al. also included polyamine as curing agent and used a hot press molding process for the preparation of compatible blends of lignin and epoxy resin [44]. However, the first works of lignin-epoxy networks correspond to the direct epoxidation reaction of lignin by reaction in basic medium at 85–90 °C with epichlorohydrin. This corresponds to the work reported by Lindström [45] who obtained gels from kraft lignin that showed a characteristic swelling behaviour of polyelectrolytic networks against changes on the pH and the ionic strength, i.e., the degree of swelling increases with the ionization grade and decreases for high salt concentrations.

**Figure 6:**
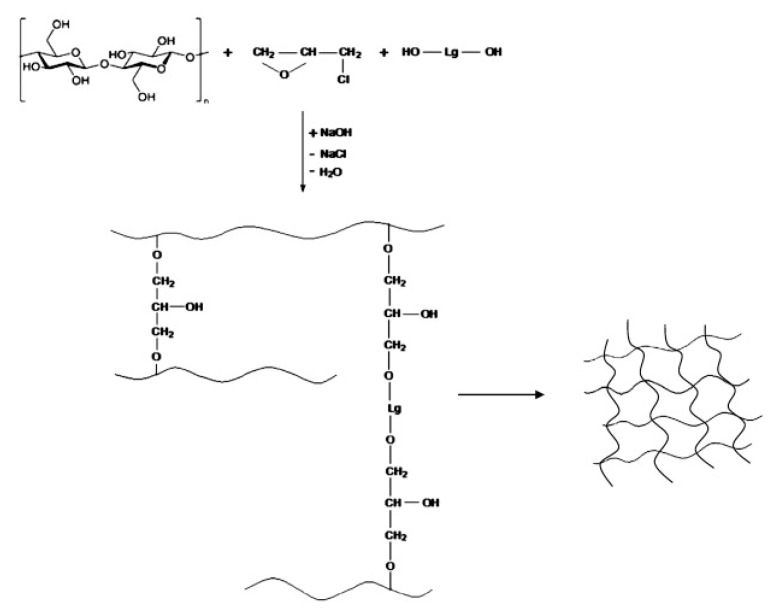
Schematic representation of the preparation of cellulose/lignin hydrogels crosslinked with epychlorhydrin. Reproduced with the permission from Ciolacu et al. [46]. Copyright © (2012) Elsevier.

Ciolacu et al. [46] made use of the reactivity of lignin and cellulose with epichlorohydrin to prepare cellulose–lignin hydrogels. These gels were easily obtained after dissolving cellulose and lignin in an alkaline solution, followed by covalent crosslinking of cellulose and lignin with epichlorohydrin (Figure 6). These superabsorbents hydrogels were evaluated for the controlled release of polyphenols. Cellulose-alkaline lignin hydrogels crosslinked with epichlorohydrin were also prepared with varying pore size for controllable fractionation of lignin by the specific adsorption and filtration of lignin with polydispersed molecular weight distribution [47].

More recently, Ciolacu et al. [48] also mixed polyvinyl alcohol (PVA) with lignin or lignin-epoxy resins to obtain hydrogels by covalent crosslinking with epichlorohydrin. Covalent crosslinking was confirmed by FTIR spectroscopy by the observation of formed ether bonds between lignin or lignin-epoxy resin and PVA. Additionally, intermolecular hydrogen bonds between PVA and lignins could be also corroborated.

A combination of above mentioned Ciolacu’s works was carried out by Huang et al. [37] employing lignin molecules as extended crosslinker, epichlorohydrin as short crosslinker, polyvinyl alcohol (PVA) as short-chain hydrophilic polymeric branches and a cellulose derivative as framework backbone. The employed long and flexible chain cellulose derivative, hydroxyethyl cellulose, allowed signifficantly high swelling (1220 g/g).

**Figure 7:**
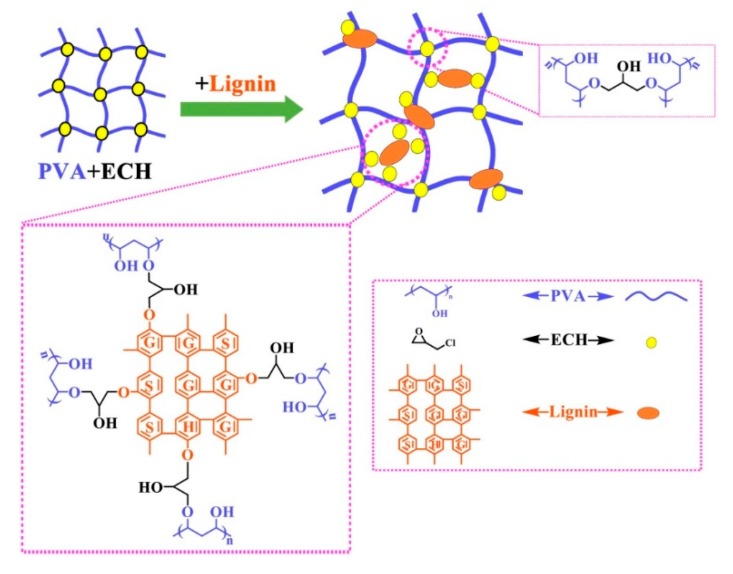
Schematic representation of the preparation of PVA/lignin hydrogels crosslinked with epychlorhydrin. Reproduced with permission from Wu et al. [49]. Copyright © (2019) Elsevier.

A simpler approach was reported by Wu et al. [49] in which PVA and lignin were crosslinked with epichlorohydrin for the preparation of a doubly crosslinked network (Figure 7), for which a lower swelling ratio (456 g/g) was measured. This work demonstrated that greater swelling properties are promoted by high molecular weights of PVA as well as high molecular weights and phenolic hydroxyl content of lignins.

A similar procedure was also reported for xanthan/lignin hydrogels [50] where chemical crosslinking was carried out in the presence of epichlorohydrin as a crosslinker agent.

The couple PVA/lignin was reported to generate chemical networks after the previous modification by amination of sodium lignin sulfonate. In addition, silver nanoparticles were in situ reduced within prepared hydrogels which enhanced the antimicrobial properties of lignin against S. aureus and E. coli [51].

On the light of Lindstrsröm’s work, lignin, acting as the polymer backbone, was directly copolymerized with more flexible crosslinker agents such poly (ethylene) glycol diglycidyl ether (PEGDGE) obtaining highly swellable lignin derivatives. A crosslinking reaction mainly occurred by an etherification reaction between PEGDGE and the phenolic OH groups of lignin due to the phenoxide nucleophile attack on the epoxide groups of PEGDGE. This crosslinker was first employed by Nishida et al. [52] for the preparation from hardwood lignin of hydrogels with a moderate swellability. This work concluded that crosslinker content did not affect swelling, due to its amphiphilicity and, consequently, swelling ability was basically ascribed to lignin.

Following the same approach, Passauer et al. [53] employed oxidatively preactivated technical lignins to be crosslinked with PEGDGE and, conversely, observed that water content of the hydrogels decrease with increasing crosslinking density. Recently, alkali lignin crosslinked with PEGDGE showed mechanical robustness in the dried state, high flexibility and being dimensionally stable in highly swelled states (up to 500% water retention), that was exploited for the development of a flexible supercapacitor [54].

Nevertheless, the most reported strategy for the preparation of lignin based hydrogels lies in the free radical polymerization, in which the phenolic hydroxyls of lignin form radicals in the presence of an initiator and react with monomers and/or polymer chains to form lignin grafted copolymers within a polymerized network, forming IPN hydrogels. In the last years, free radical polymerization with different monomers, mainly acrylamide (AAm) and N-isopropylacrylamide (NIPAAm), has been reported. In these cases the own lignin can act as crosslinking agent, or additional multifunctional monomers, such as, glutaraldehyde or *N,N*′-methylenebisacrylamide (NMBA) can also be included to promote additional crosslinking.

In the absence of an external crosslinking agent, El-Zawawy et al. [55] grafted alkaline or kraft lignin to acrylamide (AMm) and poly(vinyl alcohol) (PVA) obtaining AM-PVA-g-lignin copolymers that were then mixed with acrylamide monomer for later polymerization. More recently, the same authors [56] repeated the same formulations but adding NMBA together with AAm to achieve an extra crosslinking. As blank hydrogels, samples in which the radical initiator was not added were also studied. In the presence of the initiator, radical polymerization occurred and covalent linkages between the crosslinker NMBA and the two polymers were formed, while hydrogen bonds between graft-copolymer and monomers were only observed when initiator was not incorporated. Data showed for covalently crosslinked hydrogels a pH-sensitive swelling and higher water absorption in comparison to only physical crosslinked networks.

**Figure 8:**
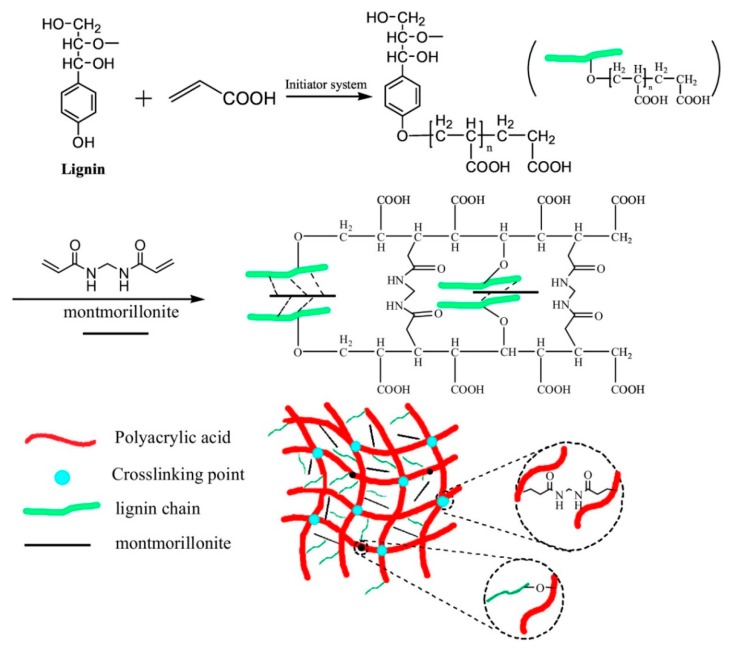
Schematic representation of the synthesis of hybrid hydrogels of lignin/PAA/montmorillonite crosslinked in the presence of NMBA. Reproduced with permission from Sun et al. [58]. Copyright © (2019) Elsevier.

Radical polymerization with other hydrophilic acrylate monomers has been also reported. Carboxymethylated lignins were graft-copolymerized with acrylic acid and the effect of lignin as crosslinking agent was analyzed in terms of water absorbance capacity [57]. Acrylic acid was also copolymerized with lignin in the presence of NMBA as external crosslinker and montmorillonite as inorganic filler, leading to superabsorbent composites with excellent adsorption/desorption performance of Cu(II) (Figure 8) [58]. For this, a redox initiator generates free radicals, which leads to the simultaneous polymerization of acrylic acid (PAA) and grafting of lignin hydroxyl groups to PAA. Later, the addition of NMBA and montmorillonite creates chemical and physical crossliking, respectively.

N-isopropylacrylamide (NIPAAm) was also employed to be polymerized with phenolated alkali lignin. Pre-activation of lignin led to higher crosslinking density and higher effect of the lignin content on the final lower critical solution temperature (LCST) of the resulted hydrogels due to the increase on the amount of reaction sites [59].

High water absorbing capacities were also measured for lignin hydrogels obtained by crosslinking a former non lignin-based copolymer; poly(methyl vinyl ether co-maleic acid) (GRANTEZ), instead of by polymerization. This is a simple and green one step process, in the solid state, and thus, as it takes place in absence of organic solvents or other reagents (such as initiators or crosslinkers) obtained hydrogels is proposed as interesting material for the industrial production. This strategy is based on the esterification reaction of hydroxyl groups of lignin with the carboxylic acids of maleic acid units. Domínguez-Robles et al. [60] analyzed the properties of the hydrogels prepared with different types of lignins (organosolv lignin, straw soda lignin and kraft lignin) for removing cationic dyes from waste water.

**Figure 9:**
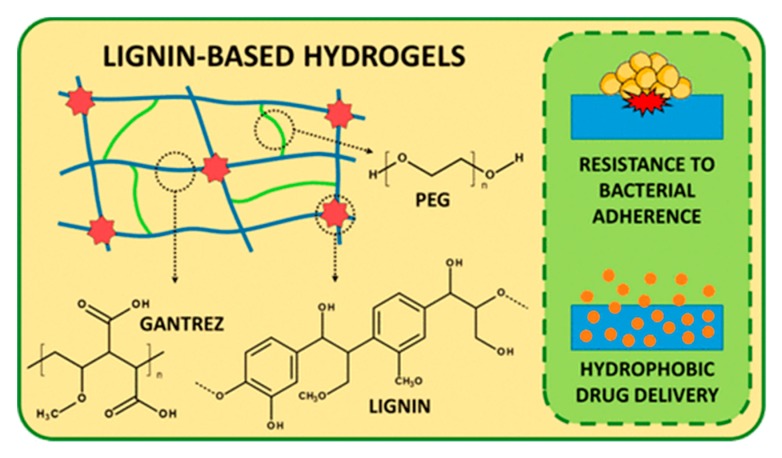
Representation of the synthetic procedure for the preparation of lignin/poly(methyl vinyl ether co-maleic acid) hydrogels crosslinked by PEG. Hydrogels showed diminished bacterial adhesion and capability for the controlled release of hydrophobic drugs. Reproduced with permission from Larrañeta et al. [61]. Copyright © (2019) American Chemical Society.

Based on the ability of poly(ethylene glycol) (PEG) to react via esterification with poly(methyl vinyl ether co-maleic acid), Larrañeta et al. [61] included poly(ethylene glycol) (PEG) in the above formulation, promoting an additional crosslinking to the network (Figure 9). In addition, they accelerated the thermal reaction proposed by Domínguez-Robles et al. by the use of microwave radiation. PEG allows great water absorption capabilities of up to 500%, successful loading, and the release of curcumin and antibacterial properties against Staphylococcus aureus and Proteus mirabilis.

**Figure 10:**
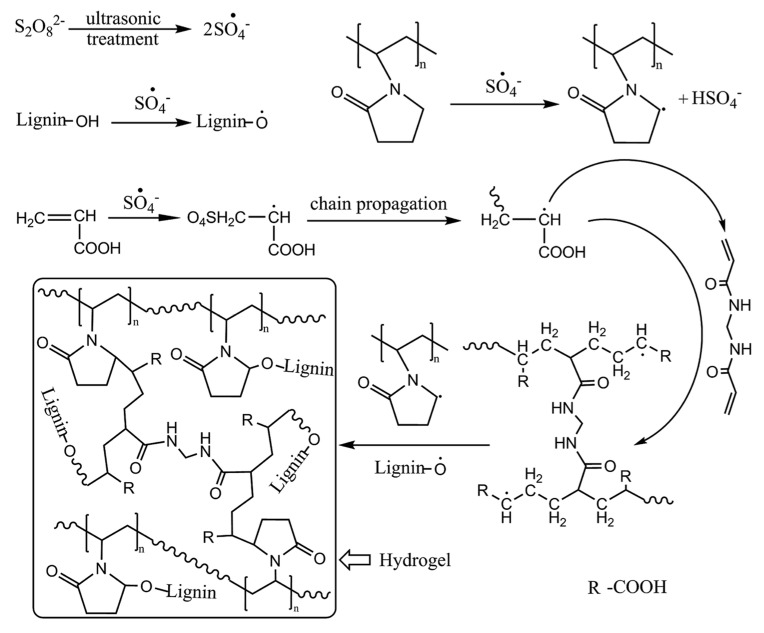
Representation of the synthesis of sodium lignosulphonate-grafted poly(acrylic acid-co-poly(vinyl pyrrolidone)) hydrogels. (Reproduced with permission from Wang et al. [62].)

Doubly crosslinked hydrogels were also prepared in lignosulphonate-grafted poly(acrylic acid-co-poly(vinyl pyrrolidone)) hydrogels by mean of the radical polymerization of acrylic acid in the presence of acid activated lignosulphonate and poly(vinyl pyrrolidone) (Figure 10). The controlled release of amoxicillin was studied as function of the pH of the medium, showing a sustained release in intestine simulated pH [62].

The main synthetic strategies described in this section are summarized in Table 1.

## 3. Applications

Lignin-based hydrogels could be considered as emerging materials with potential applications as drug delivery systems for agriculture or medicine, in water remediation applications, and in sensors.

### 3.1. Stimuli-Responsive and Smart Hydrogels

Some hydrogels present the capacity to change their volume abruptly in response to an external stimulus such as pH, temperature, or light, among others. This capacity increases the potential applications of these materials as smart and sensitive materials in aqueous solutions or in wet state. Several stimuli-responsive hydrogels based on lignin have been reported up-to-date; however, the structural limitations of this kind of hydrogels compared to other synthetic hydrogels could limit their response to a stimulus. In this case, among all the possible stimuli used to trigger their response, only hydrogels werresponsive to thermo, pH and mechanical stimulus have been reported.

In general, the incorporation of temperature sensitive monomers in the hydrogel is the most common approach for the development of thermo-responsive hydrogels. Among the possible monomers used for these formulations, N-isopropylacrylamide (NIPAAM) is the most widely used for all types of thermo-responsive hydrogels. Zerpa et al. [63] developed a thermo-responsive hydrogel by radical polymerization of lignin (kraft lignin), NIPAAM and N,N′-methylenebisacrylamide. The obtained hydrogels exhibited a critical solution temperature (LCST) between 34 and 37 °C. In addition, these hydrogels present less elastic behavior when the temperature increased, which differs significantly from the other synthetic hydrogels. Similarly, Feng et al. have reported two lignin-based hydrogels, both of them copolymerizing lignin modified with acetic acid, acetic acid lignin (AAL), and with NIPAM. In the first system *N*,*N*′-methylenebisacrylamide (NMBAm) was used as the crosslinker and H_2_O_2_ as initiator [64]. In contrast, in a second system, the OH groups of the acid were substituted with acryloyl chloride and the subsequent reaction with NIPAAM by UV photopolymerization by using 2,2-dimethoxy-2-phenylacetophenone (DMPA) as a photoinitiator [65]. All their formulations present a LCST around 30 and 32 °C.

Similarly to the thermo-responsive hydrogel, the addition of pH sensitive monomers to the formulation of a lignin-based hydrogel could be used as a highly suitable strategy to develop pH-responsive hydrogels. This kind of hydrogels could be based on the pH sensitivity of lignin at basic pH, close to 12, and the addition of ionisable counterparts such as acrylic acid, or monomers with amine groups, among others. Gao et al. [66] reported several formulations of pH responsive hydrogels obtained from kraft lignin (KL) with poly((2-dimethylamino)ethyl methacrylate) (PDMAEMA) or poly(2-(dimethylamino)ethyl methacrylate)-block-poly(ethylene oxide)-block-poly(2-(dimethylamino) ethyl methacrylate) triblock copolymer (PDMAEMA-co-PEO-co-PDMAEMA). These systems present pH-sensitivity that varies with the lignin content. In PDMAEMA homopolymer series, for weight ratios up to 1/0.33 KL/PDMAEMA a reversible soluble-insoluble (S-I) transitions were observed, this transition changes to a soluble-insoluble-soluble (S-I-S) transition, for a weight ratio higher than 1/0.67. In a triblock copolymer series, KL/triblock gel is formed for 1/0.1 weight ratio. Both series present a pH sensitivity at pH 10 were the half of the phenolic groups of the KL are ionized and consequently the hydrogen bonds formed with the PDMAEMA or the triblock copolymer are broken. On the other hand, when the pH is 2 the homopolymer, PDMAEMA is protonated and the hydrogel is also disrupted; however, the presence of the PEO block, capable to form weaker hydrogen bonds than PDMAEMA, in the triblock copolymer prevents the disruption of the hydrogel. Other example of pH sensitive hydrogels was reported by Liu et al. [67], they develop a hydrogel by free radical polymerization from lignin and acrylamide, and calcium carbonate as pore-foaming agent. The addition of pore-foaming agent increased the swelling almost 50% compared to the hydrogels without the agent. These hydrogels present a maximum swelling rate at pH = 6.8, and they reported a good stability for the hydrogels after reversibly swelling-deswelling cycles.

Some examples of dual stimuli responsive materials have been reported. Jin and co-workers [68] developed thermo and pH sensitive hydrogels from methacrylated lignosulfonate (MLS), NIPAAM and itaconic acid (IA). These hydrogels present volume phase transition temperature around the body temperature, which varies with the lignin ratio of the hydrogel. In addition, they also exhibited pH-responsivity in the range of pH 3.0 to 9.1. The lignin ratio of the hydrogels varies the swelling and the mechanical strength, decreasing the swelling and increasing the strength when MLS increases.

**Figure 11:**
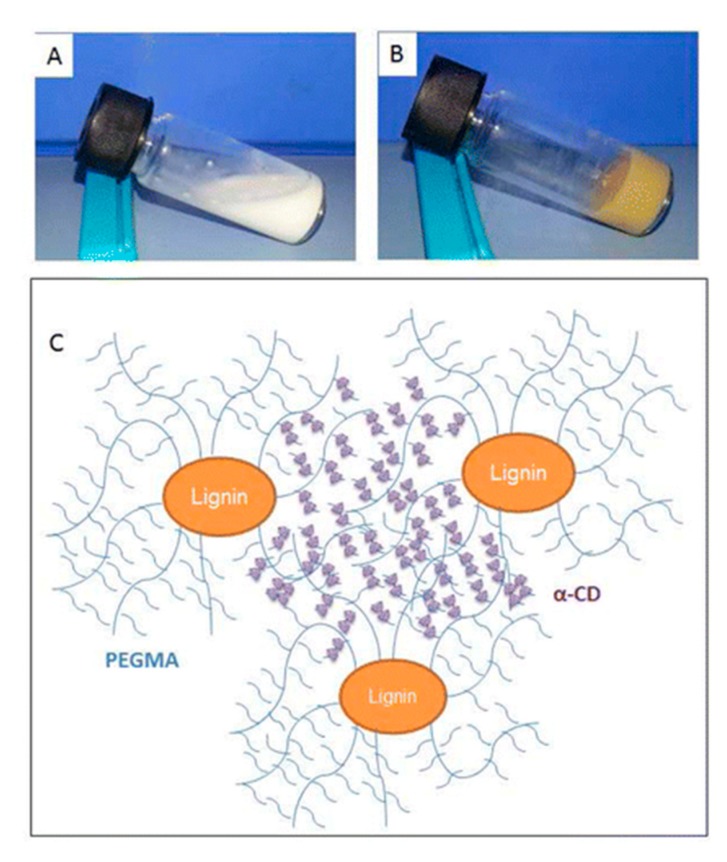
(**A**) inclusion complex suspension of PEG and α-CD and (**B**) hydrogel of PEG/lignin/α-CD and (**C**) Kai et al. proposed structure of lignin-based supramolecular hydrogel. (Reproduced with permission from Kai et al. [69]. Copyright (2015) American Chemical Society.)

An example of mechanically responsive lignin-based hydrogels was reported by Kai and co-workers [69]. It is important to notice that mechanically responsive materials are not very often described in the bibliography. These hydrogels based on lignin−PEGMA/cyclodextrin inclusion present mechanically responsive rheological properties added to excellent self-healing capabilities due to the supramolecular interactions of hydrogels by reversible host–guest inclusion complexes (Figure 11).

Recently, Huang and co-workers have developed a smart conductive hydrogel with self- healing capability. The presented hydrogels are formed by hydroxyethylcellulose, polyvinylalcohol and lignin, using borax as crosslinker [69]. The addition of lignin to hydrogels increased 34.4 times the stretchability of lignin-based hydrogel compared to one without it. Moreover, these hydrogels are capable of recovering their initial shape after four cycles of after 10,000% shear strain.

### 3.2. Hydrogels for Biomedical Applications

**Figure 12:**
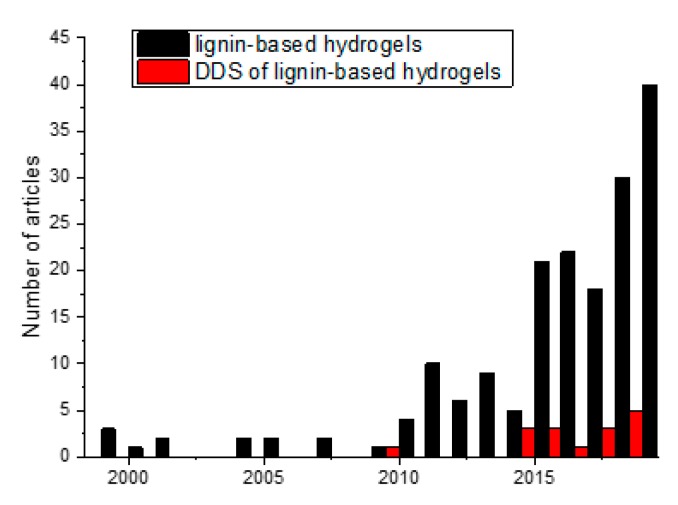
Progress of publications in (black) lignin-based hydrogels and (red) drug delivery systems based on lignin hydrogels, data obtained from Scopus.

Lignin is well-known for being inexpensive and sustainable, but when it comes to its potential in biomedical applications it is important to notice its antioxidant properties, non-toxicity, antifungal, and antibacterial properties [70]. The swelling capacity of hydrogels as a response to a trigger stimulus could be a highly desirable property to design drug delivery systems (DDS) capable to release an active agent in a concrete medium. Considering the intrinsic biological properties and the swelling capacity of lignin-based hydrogels, several DDS have been reported until now and the number of the articles devoted to this are increases every year (Figure 12).

Ravishankar and co-workers reported the fabrication of biocompatible physic hydrogels from alkali lignin and chitosan [38]. The cytotoxicity of these hydrogels was tested in vitro and in vivo, against *Mesenchymal* stem cells and zebrafish, respectively. Both studies indicate that the hydrogels were non-toxic. In addition, the authors describe a good potential applicability in wound healing since these hydrogels present a good cell attachment and proliferation of NIH 3T3 mouse fibroblast. Other example of lignin-based hydrogels for wound healing applications was reported by Zmejkoski et al. [71]. A lignin model compound from coniferyl alcohol (DHP) was used to developed hydrogels with bacterial cellulose. Several bacteria were tested to analyze the antibacterial activity of these hydrogels, such as *P*. *aeruginosa*, *S*. *aureus,* among others. The antibacterial activity of these gels is related to the DHP oligomers release, and it was observed that the antibacterial compound concentrations remain constant for 72h. The oligomers release could be highly beneficial for chronic wound healing.

The cytotoxicity of lignin-based hydrogels was also evaluated by other authors. Hydrogels obtained from kraft lignin modified with glycine-formaldehyde hyaluronan (NaHy) hydrogels have been evaluated by Musilová et al. [72]. The results indicated that lignin derivative ration does not reduce significantly the cells viability, so the resulting material could be considered as non-toxic.

Highly antibacterial lignin-based hydrogels for drug delivery have been recently reported. A logarithmic reductions in the adhesion of *Staphylococcus aureus* and *Proteus mirabilis were demonstrated for hydrogels* prepared with lignin, Gantrez S-97 (poly(methyl vinyl ether-*co*-maleic acid)) and poly(ethylene glycol) or glycerol. These hydrogels showed a swelling ratio between 24% and 40%, and this property improved the suitability of these materials as drug delivery systems. Moreover, curcumin was used as drug model, reporting a sustained delivery for up to 4 days [61].

Moreover, lignin based hydrogels have been also reported for the delivery of active agents different form drugs, such as aroma ingredients or pesticides. Hydrogels based on lignin/xanthan gum for vanillin, active aroma ingredient, release were developed by Raschip et al. [73]. On the other hand, hydrogels based on lignin and acrylic acid were successfully used for the release of pesticides. Three pesticides were loaded in them, being the loading order: cyfluthrin > paraquat > cyhalofop-butyl. The cumulative release was also higher for cyfluthrin and paraquat [74].

### 3.3. Hydrogels fo Water Remediation

The high toxicity and carcinogenicity of heavy metal ions often present in polluted water have increased the necessity of new materials capable to remediate this pollution by absorbing the contaminants. Lignin, due to its polyphenol structure and abundance, is considered a highly suitable material for this kind of applications, and some reviews had reported the effectiveness of the lignin and its derivatives as heavy metal absorbents [75,76,77,78]. These reviews reported the successful absorption of inorganic ions such as Cr(III), Cr(VI), Co(II), Ni(II), Cu(II), Zn(II), As(II), Cd(II), Hg(II) and Pb(II).

The swelling properties of the lignin-based hydrogels and the reactivity of lignin and its counterpart monomers improve the potential of these materials for being used as absorbent in water remediation applications. In addition, these hydrogels have been also tested for the absorption of organic pollutants such as dyes.

Hydrogels of bentonite/sodium lignosulfonate grafted with acrylamide and maleic anhydride presents large capacity to absorbed Pb(II), 322.70 mg/g. The adsorption of Pb(II) is preferential when the absorption is analyzed in competence with other ions such as Cu(II), Cd(II) and Zn(II) [79]. Other lignin-based hydrogels for Pb(II) absorption have been also developed such as, lignosulfonate/graphene [80] or lignin/acrylic acid [81], being capable to remove 1210 mg/g and 235 mg/g of Pb(II), respectively.

Peñaranda and co-workers described [35] an interpenetrating polymer network (IPN) of starch/acryl amide-based hydrogels and lignin capable to absorption of Cu(II) and Ni(II) from contaminated water. These hydrogels were compared with peat-based hydrogels, both of them shown a Fickean water transport mechanism, being higher the metal ion absorption capacity for lignin-based formulations.

Lignocatechol gels have been also successfully developed for metal ions absorption. Hydrogels were prepares by anchoring catechol moieties on lignin and crosslinking. The order of the selectivity among the tested ions was: Pb(II) ~ La(III) > Fe(III) > Al(III) > Ni(II) ~ Zn(II) ~ Cd(II) ~ Co(II), that is, these hydrogels present higher affinity for Pb(II) [82]. Similarly, Parajuli et al. [83] developed also a lignocatechol based hydrogels, in addition to lignophenol and lignopyrogallol-based hydrogels Au(III), being lignophenol-based hydrogel the one with the highest selectivity. The described hydrogels could act as reducing agents of ionic gold species rather than as complexing agents.

The presence of dyes and other organic pollutants in water represent a hazardous for the aquatic species. The phenolic rings present in the lignin derivatives could enhance the retention of these organic pollutants due to π-π reaction with aromatic pollutants [84]. Furthermore, the pH sensitivity of the lignin and/or its counterpart monomers could also facilitate the absorption of the contaminants by the hydrogel. Several of these studies have used methylene blue (MB) as a model dye [85,86,87].

Yu and co-workers [88] fabricated a hydrogel of lignosulfonate-g-acrylic acid with high MB adsorption capacity, 2013 mg/g. The absorption isotherm closely fits the Freundlich model, whereas kinetics could be considered of pseudo-second-order model. Lignosulfonate-based hydrogel grafted with poly (acrylic acid-*r*-acrylamide) was developed by Tang et al. [89] for adsorbing malachite green (MG) dye from water, being the absorption capacity reported for this systems 150.376 mg/g.

### 3.4. Flexible Supercapacitors and Werable Electronics

The interest for developing flexible supercapacitors (FSC) has increased parallel to the increase in the number of portable lightweight consumer devices. The use of renewable sources such as cellulose or lignin in the development of FSC coupled to the incorporation of flexible electrode/substrate material with the inherently high power density of supercapacitors have been very interesting for researchers in the last years [90,91]. However, many studies have been devoted to cellulose-based FSC [92], whereas only a few studies have reported lignin-based materials for FSC [93,94]. An assembly of lignosulfonate/single-walled carbon nanotube hydrogels was used as electrodes for the formation of FSC by using cellulose hydrogels as electrolyte separators. The reported FSC present high specific capacitance, 292 F/g at the current density of 0.5 A/g, and excellent energy density of 17.1 W·h/kg [95].

**Figure 13:**
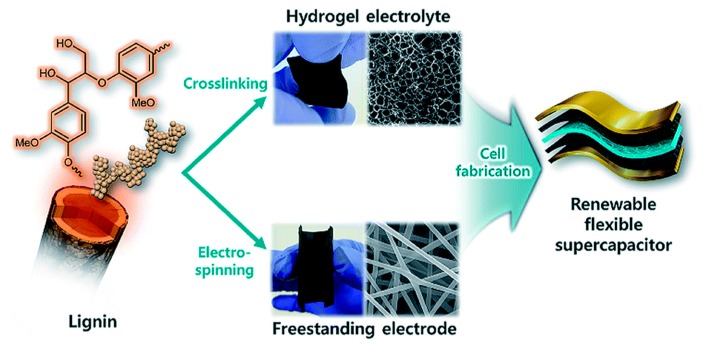
Scheme of the fabrication of an FSC based on an electrolyte of lignin hydrogel electrolyte and electrode of lignin/PAN/carbon nanofiber fabricated by Park et al. Reproduced from Ref. [54] with permission from The Royal Society of Chemistry. Copyright (2019) The Royal Society of Chemistry.

Similarly, Park and co-workers designed an FSC wherein with the lignin was present in the electrolyte and in the electrode. The electrolyte was fabricated with lignin crosslinked with poly(ethylene glycol) diglycidyl ether whereas the electrode was obtained by electrospining of lignin/polyacrylonitrile (Figure 13). The reported FSC presented a high capacitance of 129.23 F g^−1^, a maximum energy 4.49 W h kg^−1^and power density of 2.63 kW kg^−1^ [54].

A lignin was used as a crosslinker for the development of hydrophilic polyether-based polyurethane (HPU) hydrogels to fabricate flexible films for a wearable electronic. The incorporation of the lignin crosslinker increased the mechanical properties, by increasing significantly the fracture energy and Young’s modulus. It is important to notice the processability of these materials, being tested by Oveissi et al. various techniques, such as fiber spinning, casting, and 3D printing [36]. Table 2 summarized the main applications of lignin-based hydrogels described in this review

## 4. Future Perspective and Conclusions

The abundance of lignin, since it is the main by-product of the pulp and paper industries, has dramatically aroused the interest in lignin-based materials. In an industry increasingly aware of environmental problems, the valorisation of once being listed as a production waste by developing new materials based on it has presented a potential new business field. Until recently, the main efforts incorporating lignin-based materials have been focused on the fabrication of thermoset and polyurethanes. However, although the research in these materials remains highly important in the field, every year, an increasing number of research groups are developing lignin-based hydrogels. The results being reported are very encouraging, which suggests that they may have wide applications as biomaterials, drug delivery systems, or in water remediation.

## Figures and Tables

**Table 1:** Main synthetic strategies described in the bibliography for the development of lignin based hydrogels.

Lignin Role	Matrix	Crosslinker	Lignin Interaction	Ref
Lignin as biomass	Polymerized acrylamide	N,N′-methylenebis(acrylamide) (NMBA)	H-bonding	[35]
	Polyurethane	−	H-bonding	[36]
	Hydroxyethyl cellulose and polyvinyl alcohol	Borax	H-bonding	[37]
Lignin as crosslinked unit	Chitosan	Lignin	Electrostatic	[38]
	Phenol/lignin/formaldehyde Lignin-tannin-formaldehyde	Formaldehyde	Chemical	[41]
	Lignin–phenol–resorcinol	Glutaraldehyde	Chemical	[40]
	Lignin and epoxy resin	Polyamine	Chemical	[44]
	Lignin	Epichlorohydrin	Chemical	[45]
	Cellulose and lignin	Epichlorohydrin	Chemical	[46,47]
	Polyvinyl alcohol and lignin or lignin-epoxy	Epichlorohydrin	Chemical/H-bonding	[48,49]
	Lignin	Poly (ethylene) glycol diglycidyl ether	Chemical	[52,53,54]
	Acrylamide-Polyvinyl alcohol-graft-lignin copolymers	−/NMBA	Chemical	[55,56]
	Xanthan and lignin	Epichlorohydrin	Chemical	[50]
Lignin as crosslinking agent	Chitosan and polyvinyl alcohol	lignin	H-bonding/ electrostatic	[39]
	Cellulose derivatives and polyvinyl alcohol	Lignin/epichlorohydrin	Chemical	[37]
	Polyvinyl alcohol	Aminated li gnin	Chemical	[51]
	Polymerized acrylic acid	Lignin/lignin and NMBA	Chemical	[57,58]
	Polymerized N-isopropylacrylamide	Lignin	Chemical	[59]
	Poly(methyl vinyl ether co-maleic acid) (GRANTEZ)	Lignin/Lignin and PEG	Chemical	[60,61]
	Polymerized acrylic acid and poly(vinyl pyrrolidone)	Lignin	Chemical	[62]

**Table 2:** Main applications of lignin-based hydrogels.

Smart Hydrogels		
**Active Hydrogels**		
	Thermoresponsive	[63,64,65]
	pH-responsive	[66,67]
	Dual (Thermo and pH)	[68]
	Mechanically responsive	[69]
Biomedical applications		
	Antibacterial	[38,72]
	Drug release	[61]
	Aroma	[73]
	Pesticide release	[74]
Water remediation		
	Metals	[35,75,76,77,78,79,80,81,82,83]
	Dyes	[85,86,87,88,89]
Supercapacitators and weareble		
	Supercapacitators	[54,93,94,95]
	Wearable electronic and 3D printing	[36]
